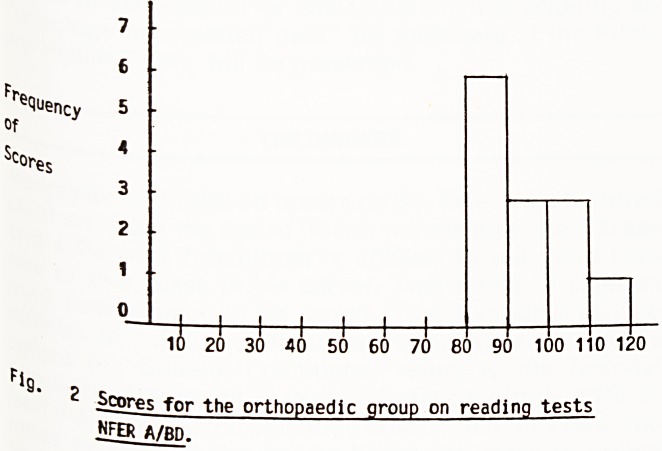# Some Educational Implications of Childhood Cancer

**Published:** 1988

**Authors:** Chris Colburn

**Affiliations:** Bristol Children's Hospital


### Some Educational Implications of Childhood Cancer

**Chris Colburn**

Bristol Children's Hospital

### INTRODUCTION

Research into the social and psychological effects of sickness on children with such chronic diseases as asthma, diabetes, haemophilia and cystic fibrosis is inconclusive; some studies (Olch, 1971; Gayton and Friedman, 1974) indicate that these children often fall short of their educational potential, but others (Burton, 1974; Tavormina et al, 1976) suggest that children develop coping strategies, and tend to perform at least averagely in academic tasks. A similar diversity is to be found in the literature concerning social adjustment (Brown, 1979; Knowles, 1971; Swift, Seidman and Stein, 1967).

Most of the research relating to children with cancer has taken place in America, and although there is some measure of disagreement between the findings of the various studies, there is a general consensus that children in school after treatment for cancer face an increased likelihood of experiencing difficulties (Eiser, 1980; Green, 1975; Spinetta, 1980). These potential problems include:

a) Poor school attendance b) Behavioural disturbance c) Non-fulfilment of academic potential.

This piece of research, which was undertaken as part of a Masters Degree in Education, was designed to assess the re-integration into school and subsequent classroom performance of a small group of children who had suffered from cancer.

### The Sample

The sample of children was identified by the following criteria:

1. Ages between 4 and 11 years (Primary school age).

2. Attendance at school before hospitalization.

3. Living in the same county.

The final sample consisted of 14 children (8 boys and 6 girls) with a range of tumours and leukaemia. Each member of the experimental group was matched with a child who had been a patient at a hospital in the same Health Authority, who had suffered from a variety of orthopaedic conditions. Although these conditions are not comparable in terms of life-threat, most of the control sample had undergone major surgery which was debilitating for a considerable length of time. Individual matching was effected on the following criteria:

1. Age

2. Sex

3. Date of hospitalization

The use of a control group was to provide a basis for comparison, so that difficulties experienced by children with cancer could be differentiated from those experienced by another group of hospitalized children. Their inclusion in the study also aided confidentiality. The experimental and control samples consisted of fourteen children each, aged between 7.17 years and 11.5 years. In each group there were eight boys and six girls. The children had been hospitalized for between 3 and 10 weeks. The mean length of hospitalization for the experimental sample was 6.8 weeks, and for the control group, 5.5 weeks. Time since diagnosis ranged between 1 year and 4½ years. All the children in the orthopaedic group had undergone surgery, and the children in the oncology group had received radiotherapy and chemotherapy, and two had also undergone surgery. The variety of schools which the children usually attended reflected the diversity of educational provision in the county concerned.

### METHODS

Several methods of investigation were employed, these included questionnaires, interviews, rating scales and a standardized test of academic performance.

Great care was taken in designing questionnaires and interviews not to refer to the particular circumstances of the child's hospitalization.

### RESULTS

The results of the study can be presented in four main sections:

a) *School Attendance*
Information regarding attendance at school since hospitalization was gained on visits to the schools. In the experimental group ten children (71%) were reported to have good attendance (80–100 percent), three children (21%) attended fairly well (65–80 percent) and one child had poor attendance (less than 65 percent). This compared with 85% good attendance and 14% fairly good attendance in the control group.

Table 1 shows how these figures relate to length of hospitalization and time since hospitalization.

It would seem that for this sample, neither the length of hospitalization, nor the time since hospitalization are determining factors in current attendance.

b) *Social Adjustment*
Indications of how children re-adjust to school after hospitalization were made via several sources:—

1. The Behaviourl Questionnaire (adapted version of the Deasy-Spinetta Questionnaire).
2. The Bristol Social Adjustment Guide.
3. The interviews.

Several studies (Eiser, 1980; Green, 1975), and particularly Spinetta 1980) have identified behavioural differences between cancer patients and other children. The modified version of Spinetta's Behavioural Questionnaire which illuminated these differences in the American research, gave the following results (Table 2).

In both groups, the majority of children were rated as stable on the Bristol Social Adjustment Guide. However, where poor adjustment was detected, orthopaedic children tended to over-react and oncology children tended to under-react. Table 3 gives the BSAG ratings.

This tendency is borne out by comments recorded at teachers' interviews – three teachers said that their

**Table 1**

**Relationship between recent attendance, length of hospitalization and time since hospitalization**

| Child | Attendance | | Length in Hosp$^n$ (in weeks) | | Time Since Hosp$^n$ (in years) | |
|---|---|---|---|---|---|---|
| | Oncology | Orthop | Oncology | Orthop | Oncology | Orthop |
| 1 | Good | Good | 4 | 4 | 2 | 2 |
| 2 | Good | Good | 7 | 6 | 2 | 2 |
| 3 | Poor | Fair | 8 | 6 | 2 | 2 |
| 4 | Good | Fair | 7 | 5 | 2 | 2 |
| 5 | Good | Good | 4 | 4 | 3 | 2 |
| 6 | Fair | Good | 6 | 3 | 4·5 | 4.5 |
| 7 | Good | Good | 8 | 10 | 1·5 | 1.5 |
| 8 | Good | Good | 4 | 3 | 2 | 2 |
| 9 | Good | Good | 6 | 3 | 1 | 1 |
| 10 | Fair | Good | 9 | 8 | 2 | 1 |
| 11 | Good | Good | 12 | 9 | 3 | 2 |
| 12 | Good | Good | 6 | 3 | 3 | 2.5 |
| 13 | Fair | Good | 4 | 3 | 1 | 2 |
| 14 | Good | Good | 10 | 12 | 3.5 | 2 |

**Table 2**

**Behaviour Questionnaire: Comparisons of Experimental Group and Control Group**

| | % Oncology Group | % Orthopaedic Group |
|---|---|---|
| Lack of Concentration | 14 | 14 |
| General Learning difficulties | 14 | 14 |
| Problems with reading | 21 | 36 |
| Problems with maths | 42 | 57 |
| Problems with sequencing activities | 21 | 36 |
| Underactive and lethargic | 50 | 0 |
| Anxious | 50 | 29 |
| Self-conscious | 64 | 42 |
| Unexpressive | 42 | 7 |

**Table 3**

**Under- and over-reaction ratings on the BSAG**

| | % Oncology Group | % Orthopaedic Group |
|---|---|---|
| *Under-reaction Ratings* | | |
| Stable | 64 | 71 |
| Mild underreaction | 21 | 21 |
| Appreciable Underreaction | 0 | 0 |
| Maladjusted Underreaction | 14 | 7 |
| *Over-reaction Ratings* | | |
| Stable | 85 | 78 |
| Mild Overreaction | 14 | 7 |
| Appreciable Overreaction | 0 | 0 |
| Maladjusted Overreaction | 0 | 14 |

orthopaedic child was attention-seeking, and could be a nuisance; several others commented on lack of concentration, noisiness and naughty behaviour. Teachers of oncology children commented on the quietness of the children, their willingness to help, and their need to do well in class.

The incidence of self-consciousness was high for both groups. For many children, a change in body image has taken place and this may be reflected in the scores. Many teachers noted that the orthopaedic child in their class was physically different – perhaps walked with a limp, or was noticably clumsy or 'gawky'; this was without exception linked to the child's operation or condition.

Most children in the oncology group will also have been through physical changes during treatment, although this is usually a temporary change. Nevertheless, the adaptation in body image in order to cope with hair loss, scars, weight gain or loss, and so on, is quite significant. Children undergoing cancer treatment are usually expected to return to school wearing a wig or scarf until the hair grows again; and the change in body size may not stabilize for some time. Thus it is not surprising that the majority of cancer patients and a large minority of orthopaedic patients are rated as behaving in an overly self-conscious way.

c) *Classroom Performance*

Both groups of children were assessed in classroom achievements (academic and non-academic) on the Teachers Rating scale. Children were rated on the scale: very much above average/ above average/ average/ below average/ very much below average in each activity. These were translated into scores where 'very much above average' scored 5 points, and 'very much below average' scored 1. The results are shown on Table 4.

The Teacher Rating was a subjective assessment, and it must be borne in mind that standards in the schools varied, and an average achievement in one classroom could not be said to be the same for another.

The one objective assessment of academic performance was the reading test. NFER tests A or BD were used according to the ages of individual children. Children in the samples had not used the test before.

Scores for the experimental group ranged from 83 to 112. The mean score was 98.0. For boys only the mean score was 96.9, for girls 99.8.

Scores for the control group ranged from 82 to 111, the mean score was 93.4; the boys' mean score was 91.4, and the girls' was 96.6. A child with average reading ability in the general population would be expected to score around 100.

**Table 4**

**Overall average scores of experimental and control groups from the Teachers Rating Scale, where 5 is the highest score and 1 is the lowest**

| | *Oncology Group* | *Orthopaedic Group* |
|---|---|---|
| Reading | 3.0 | 2.9 |
| Spoken language | 3.0 | 2.8 |
| Written language | 2.9 | 2.4 |
| Maths/number work | 2.6 | 2.4 |
| Art and craft | 3.2 | 2.9 |
| P.E. and sport | 2.7 | 3.0 |
| Behaviour | 3.6 | 3.3 |

The following histograms, Figures 1 and 2, display the frequency distribution of reading scores.

Fig. 1 Scores for the oncology group on reading tests NFER A/BD.

Fig. 2 Scores for the orthopaedic group on reading tests NFER A/BD.

d) *Teachers' Attitudes*

Teachers' attitudes are seen to be of central importance to children's re-integration into school post-diagnosis. Most teachers had been informed about the child's condition, how much time had been missed from school previously, and an idea of the treatment used. Two Headteachers said they did not have sufficient information: both children had transferred to other schools after hospitalization. Several Heads complained that they had never received any medical information from the hospital or schools doctor, and that they relied totally on parents. Three teachers mentioned that they had not known about the risks involved if the child contracted measles or chicken pox. This vital information had been omitted by parents, or not passed on within the school.

Several teachers expressed the need for a channel of communication to be established with the hospital for queries relating to the children. These queries fell into two main categories, and the teachers felt they could not always ask the parents about the issues raised:

1) How much should teachers 'push' the children into taking part in activities, for example should they go out at playtime? Should they be made to do P.E.? How should the children be disciplined? Several teachers said that if they disciplined the child in the same way as the rest of the class, the child might cry, or become withdrawn and pale. Teachers percieved a different reaction in the ex-cancer patient than other children in the class.

2) How much can teachers expect from the children in terms of academic achievement? Had the treatment damaged part of the brain? Could the trauma of life-threat have affected the child's adjustment and motivation?

The teachers of children in the experimental group were thus aware of a "differentness" of children with cancer. They encouraged parental involvement, and relied on parents for information about medical matters. However, parents of these children were often described as being overprotective.

### DISCUSSION

The general conclusion that can be drawn from this study (which is limited by the smallness of the sample), is that primary school age survivors of childhood cancer share some problems common to other children after hospitalization, but that they face further potential difficulties which can be identified as being specific to the condition. Common post-hospitalization problems include short or long-term adjustment difficulty; short-term academic lag, and longer term problems with such areas of the curriculum which depend on previously understood concepts, for example in mathematics. Additional potential problems identified as being specific to cancer patients are: poor school attendance, lethargy, self-consciousness and anxiety, and under-reactive poor social adjustment.

The study indicates that there are areas of concern with regard to the education of children with cancer. Further research is necessary to clarify whether the conclusions of this study are common to other geographical locations, and across the age range. It also points the way to certain improvements which teachers and others involved in the care and education of children with cancer could implement.

Firstly, channels of communication between hospital medical staff, hospital and home teaching services, and schools, could be made more effective. Parents must retain their current authority, but where possible, they should be encouraged to allow access to relevant information to schools by the usual interdisciplinary methods. Many parents would welcome this approach, as it would relieve them of the burden of conveying medical messages themselves, although they should be included in the process at every level. Access to information about cancer generally, and the effects of the illness and its treatment, on individual pupils, would help teachers understand the condition, and thereby create a foundation from which he or she could handle potentially difficult classroom situations involving the cancer patient or his peers. Teachers' understanding of the child's absences, side effects of therapies and the need for a balance of realistic academic expectations should enhance the child's chances of positive school experiences, by reducing anxiety and boosting self-esteem.

Secondly, hospital and home teaching staff should be made aware of the implications of chronic illness on certain areas of the curriculum. The proportion of children assessed at a below-average level in mathematics is significant (57% and 42% in the two groups of hospitalized children). Hospital and home tutors are frequently in a one-to-one or small-group teaching situation, which could be utilized to assess difficulty and make up any deficit.

Improvements in these two areas may help children with cancer to lead a normal life at school.

### REFERENCES

BROWN, B. (1981) Beyond Separation. *In.Fletcher, B.*

BURTON, L. (1975) The Family Life of Sick Children. *Routledge and Kegan Paul.*

EISER, C. (1980) How Leukaemia Affects a Child's Schooling. (1980) *Br.J. of Social and Clinical Psychology.* Vol. 19, no.4, pp 365–368.

FLETCHER, B. (1981) Psychological Upset in Post-Hospitalized Children: A Review of the Literature. *Maternal-Child Nursing J.* Vol 10, no. 3, pp 185–195.

GAYTON, W. F. and FRIEDMAN, S. B. A. (1973) A Review of the Psychosocial Aspects of Cystic Fibrosis. *Am.J. of Diseases in Childhood.* **126**, 856–59.

GREEN, P. (1975) The Child with Leukaemia in the Classroom. *Am.J. of Nursing.* **75**, 86–87.

KNOWLES, H. C. (1971) Diabetes Mellitus in Childhood and Adolescence. The Medical Clinics of North America, Vol. 55, pp 1007–1019.

OLCH, D. (1971) Effects of Hemophilia Upon Intellectual Growth and Academic Achievement. *J.Genet.Psychol.* Vol. 119, p 63-74.

SPINETTA, J. J. and DEASY-SPINETTA, P. (1981) (Eds.) Living with Childhood Cancer. St. Louis, Missouri. C. V. Mosby.

SWIFT, C. R., SEIDMAN, F. and STEIN, H. (1967) Adjustment Problems in Juvenile Diabetes. Psychosomatic Medicine. Vol. 29, pp 555–571.

TAVORMINA, J. B., KASTNER, L. S., SLATER, P. M. and WATT, S. L. (1976) Chronically Ill Children – A Psychologically Deviant Population? *J. of Abnormal Child Psychology.* Vol. 4, pp 99–111.

## Figures and Tables

**Fig. 1 f1:**
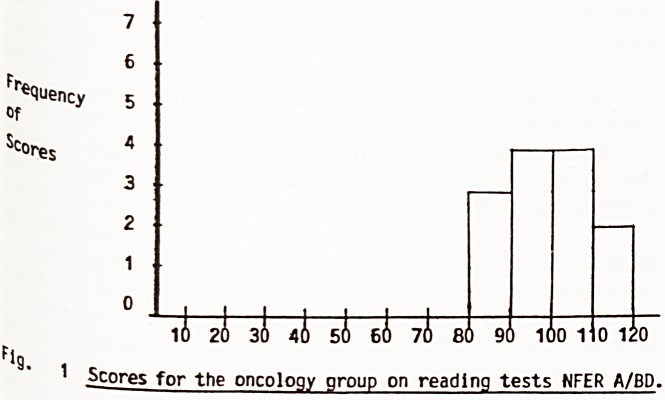


**Fig. 2 f2:**